# Inflammatory Bowel Diseases Before and After 1990

**DOI:** 10.1016/j.gastha.2022.08.001

**Published:** 2022-08-10

**Authors:** Brindusa Truta, Ferdouse Begum, Lisa Wu Datta, NIDDK IBD Genetics Consortium, Steven R. Brant, Judy H. Cho, Richard H. Duerr, Dermot B.P. McGovern, John R. Rioux, Mark S. Silverberg, Steven R. Brant

**Affiliations:** 1 Division of Gastroenterology, Harvey M. and Lyn P. Meyerhoff Inflammatory Bowel Disease Center, Johns Hopkins University School of Medicine, Baltimore, Maryland; 2 Department of Epidemiology, Johns Hopkins Bloomberg School of Public Health, Johns Hopkins University, Baltimore, Maryland; 3 Division of Gastroenterology and Hepatology, Department of Medicine, Rutgers Robert Wood Johnson Medical School, The Rutgers Crohns and Colitis Center of New Jersey, New Brunswick, New Jersey; 4 Department of Genetics and the Human Genetics Institute of New Jersey, Rutgers University, New Brunswick, New Jersey

**Keywords:** IBD, Genetic, Smoking, Environmental Risks

## Abstract

**Background and Aims:**

Inflammatory bowel disease (IBD) is caused by interaction of genetic and environmental risk factors. We evaluated potential determinants of the post-1990 increased incidence in North America.

**Methods:**

Using fitted generalized linear models, we assessed clinical features, smoking and genetic risk scores (GRS) for Crohn’s disease (CD) and ulcerative colitis (UC) in the National Institutes of Diabetes, Digestion and Kidney Diseases IBD Genetics Consortium database, before and post 1990.

**Results:**

Among 2744 patients (55% CD, 42.2% UC), smoking status and GRS were the main determinants of diagnosis age. After 1990, smoking at diagnosis declined significantly in both UC and CD (34.1% vs 20.8%, *P* < .001, and 14.7% vs 8.7%, *P* = .06, respectively). In UC, ex-smoking increased (9% vs 15%, *P* < .001), and nonsmoking rates remained unchanged, whereas in CD, ex-smoking remained unchanged. CD-GRS and IBD-GRS were significantly associated with young diagnosis age, Jewish ethnicity, IBD family history, and surgery. CD-GRS showed a borderline significant decrease (*P* = .058) in multivariate analysis post 1990 but only when excluding surgery in the model; surgery significantly decreased post 1990 in both CD and UC. CD-GRS inversely correlated with smoking at diagnosis (*P* < .001) suggesting that, in the presence of smoking, CD may only require a low genetic risk to develop.

**Conclusion:**

Significantly increase in ex-smoking correlates with UC incidence post 1990. Conversely, smoking risk decreased significantly post 1990 despite rising CD incidence. CD-GRS likewise trended to decrease post 1990 only when not accounting for a significant decrease in CD surgery. We therefore deduce that unaccounted risk factors (eg, dietary, obesity, antibiotic use, improved hygiene, etc.) or greater detection or presence of mild CD may underlie post-1990 increased CD incidence.

## Introduction

Inflammatory bowel disease (IBD), comprising Crohn’s disease (CD) and ulcerative colitis (UC), is a chronic condition that decreases quality of life through debilitating diarrhea, bloody stools, and abdominal pain. After the emergence in the Western world in the early 20th century, IBD incidence and prevalence accelerated parallel with societal development, reaching the highest values in North America, Europe, and Australia.^1^, ^2^, ^3^ In the United States, IBD affected 1.8 million adults in 1999 and 3 million adults in 2015.^4^

Both genetic and environmental IBD risk factors have been identified.^5^, ^6^, ^7^ Within a given geographic area, IBD family history remains the strongest risk factor.^8^ The risk of IBD for a person who has a first-degree relative with CD or UC is 10-fold and 4-fold higher, respectively, than the general population.^9^, ^10^, ^11^ The first risk gene identified by linkage studies for CD was *NOD2*, with 3 loss-of-function mutations primarily responsible for the genetic association.^12^^,^^13^ Subsequent genome-wide association studies identified multiple IBD risk genes, *TNFSF15* (identified in Japan), followed by *IL23R*, *ATG16L1*, *IRGM*, and *PTGER4* for CD, and established the human leucocyte antigen locus as dominant in UC. Genome-wide association studies meta-analyses and replications have identified over 260 genetic risk loci for IBD, and most loci contain genetic risk variants found commonly among European ancestry populations.^12^, ^13^, ^14^, ^15^, ^16^, ^17^, ^18^, ^19^, ^20^, ^21^, ^22^ Aside from *NOD2* and *IL23R* with greater than 2-fold increased risk for single-risk variants (and approximately 20-fold for *NOD2* mutation homozygotes), the remainder of the commonly inherited IBD risk variants individually contribute very modestly to IBD risk, with odds ratios less than 1.3 to just above 1.0 for most loci. A high genetic risk score (GRS), determined by the total number of common risk variants, each weighted by their contribution to IBD relative risk,^14^ was associated with younger age at diagnosis and involvement of the terminal ileum in CD.^23^ However, except for the highly penetrant Mendelian forms of very early-onset IBD,^24^ genetic risk variants show low penetrance and hence have been predicted to require interaction with uncharacterized environmental risk factors for IBD to develop.^25^, ^26^, ^27^

Environmental risk factors identified in IBD include smoking, appendectomy, oral contraceptives, diet and obesity, breast feeding, infections, antibiotics, food additives, and childhood hygiene.^25^, ^26^, ^27^, ^28^, ^29^, ^30^ Among these, the best-characterized and most rigorous, measurable environmental risk factor is cigarette smoking.^27^, ^28^, ^29^, ^30^ Current smoking confers a 2-fold increase in CD compared to nonsmokers and is associated with a greater likelihood of aggressive disease including need for surgery. Conversely and paradoxically, nonsmokers have an increased risk of UC as compared to smokers. Furthermore, smoking cessation is associated with a significant increase in UC incidence, and when compared to never smokers, this effect can last up to 10 years after smoking cessation.^29^

The greatest environmental risk factor, however, is living in a Western industrialized country.^5^ Migration studies have shown that for the same ancestral population, IBD risk increases greatly when ethnic groups move from low-incidence, non-Western, and less-industrialized countries to the high-incidence, Western industrialized countries.^31^, ^32^, ^33^, ^34^, ^35^, ^36^ Within a given country, industrialization and concurrent lifestyle changes (improved environmental hygiene, food refrigeration, and dietary changes including greater sugar intake and food additives such as emulsifiers) have correlated with increased IBD incidence.^37^, ^38^, ^39^

The most rigorous evaluation of IBD incidence in the United States, determined multiple times over several decades, has been in Olmsted County, Minnesota, by epidemiologists at the Mayo Clinic.^40^ CD incidence rose from 6.9 per 100,000 person-years in the 1970 to 9.0 in the 1990s and 10.7 during the first decade of the current century (2000–2009). UC incidence remained relatively stable across the last 3 decades of the twentieth century (9.2, 10.2, and 10.2) only to increase to 12.2 in the first decade of the current century.

We hypothesized that if the rise in IBD cases in the 1990s and 2000s was due to novel environmental causes or improved detection of mild cases, then IBD genetic risk among cases would decrease. To address this hypothesis, we explored demographic, environmental, and genetic risk factors in a large North American IBD database, the well-characterized National Institutes of Diabetes, Digestion and Kidney Diseases IBD Genetics Consortium (IBDGC). We also examined temporal trends in age at diagnosis, disease severity, and their determinant factors.

## Materials and Methods

### Study Population

This is a case-control study on white, non-Hispanic, participants of European descent, carrying a confirmed diagnosis of CD, UC, and IBD-unclassified (IBD-U) registered with IBDGC. The IBDGC cohort was established in 2003, when 6 genetic research centers (GRCs) in the United States and Canada began recruiting patients with confirmed CD, UC, or IBD-U, for IBD gene discovery.^41^ Recruitment was unbiased by IBD family history or other clinical factors except that, during the initial 2 years of recruitment (2003–2004), UC proctitis was excluded, and UC cases were preferentially those with extensive disease. IBD cases recruited at each center had their diagnoses of CD, UC, and IBD-U confirmed by chart review, as per the IBDGC phenotyping manual (https://ibdgc.uchicago.edu/files/IBDGC_Phenotyping_Manual_2019-10.pdf). The information was collected one time only, at the time of recruitment.

Data for each patient included demographics, family history, smoking history, and details pertinent to the date of diagnosis, extent and location of the disease, and extraintestinal manifestations. Blood was drawn for DNA extraction for genotyping. Healthy controls in the database were unrelated individuals without IBD. Spouses, significant others, and same-gender friends were targeted as controls, and the general population was also recruited.

### Inclusion Criteria

The IBDGC centers recruited patients of all ethnicities. However, this study was limited to white, non-Hispanic participants of European descent, carrying a confirmed diagnosis of CD, UC, or IBD-U, given the focus on IBD-GRS as a major predictor variable. GRS in IBD has only been validated in European ancestry populations with the majority of genetic loci and variants not established or having differential risk among non-whites.^42^

### Exclusion Criteria

We excluded patients diagnosed under age 6 (very-early-onset IBD) and those with missing or incomplete genotype data. Multiple related individuals were excluded (eg, only 1 person per family was included). Children were not included as controls.

### Definition of Variables

*Date of diagnosis* refers to the year when a definitive diagnosis of any IBD phenotype was first made.

*Smoking at diagnosis* is defined as smoking, on average, at least 1 cigarette daily for at least 3 months before diagnosis.

*Ex-smoker at diagnosis* is defined as smoking stopped at least 3 months before diagnosis and was not smoking at study entry. Nonsmokers (cases and controls) were individuals who smoked fewer than 100 cigarettes in their lifetime.

*Family history of IBD* is a history of CD, UC, or IBD-U in a first- or second-degree relative, self-reported by the study subject.

*Jewish ethnicity*, in studies in North America and Western Europe, has been consistently associated with a significantly greater disease prevalence and IBD family history, hence the need for adjustment. *Jewish ethnicity* was self-reported as Jewish ancestry in grandparents.

*Characteristics of disease* comprised disease extent (Montreal classifications L1, L2, L3, and L4 for CD; E1, E2, and E3 for UC), behavior (B1, B2, and B3), perianal disease, extraintestinal manifestations (EIMs), or history of bowel resection. EIMs were determined as per the IBDGC phenotyping manual.^41^

*Surgery* was limited to intestinal resections: total colectomy for CD and UC patients and segmental small-bowel or large-bowel resections for CD patients.

#### Genetic Risk Score

All patients and healthy controls were genotyped with the custom Immunochip array (Illumina, San Diego, CA).^13^ Based on the weighted sum of all risk alleles, we calculated a composite GRS for each individual, using the R program Mangrove.^14^ We used the list of lead IBD-associated single-nucleotide polymorphisms (SNPs) and their odds ratios for CD, UC, and IBD for each independent locus, and their risk allele frequency estimations in European ancestry individuals, as reported by Liu et al.^15^ Because our study was limited to individuals of European descent, we limited SNPs to those established as significant (with *P* < 5 × 10^−8^ association evidence) in Europeans for each locus. We selected 145 CD SNPs, 89 UC SNPs, and 162 IBD SNPs for a given blood sample of a patient or control individual. We included the 3 common *NOD2* loss-of-function mutations (rs2066844, rs2066845, and rs2066847) for the “CD” and “IBD” groups.

### Ethical Considerations

The ethical boards of each GRC approved the study. All patients included in this study gave written informed consent including for a genetic research study.

### Statistical Analysis

To determine the temporal changes that occurred in each of the 3 disease phenotypes and the determinant factors associated with these changes, we analyzed (1) disease characteristics (age of onset, disease extent, and severity), (2) smoking habits, and (3) GRS depending on when each patient was diagnosed (1940–1989 [“pre-1990”] or 1990–2011 [“post-1990”]). Distributions of study variables were compared by disease status (ie, patients vs healthy controls) and by diagnosis (CD, UC, and IBD-U). Data are presented as frequencies and percentages for categorical variables, means with standard deviations for continuous normally distributed variables, and medians with 25th–75th percentiles for continuous skewed variables. To test associations between continuous variables and disease status, we used 1-way analysis of variance. To test associations between categorical variables and disease status, we used Pearson’s Chi-squared test.

To assess predictors of age at diagnosis, for the entire sample and separately for CD and UC, we used linear regression models with robust variance and bootstrapped 95% confidence intervals (CIs). To assess risk difference in current smoking by decade (dichotomized as 1940–1989 and 1990–2011), we fitted generalized linear models for binomial family, and additionally, we adjusted for age at diagnosis and GRS score. We fitted quantile regressions to compare median GRS scores by time period (dichotomized as 1950–1989 and 1990–2011); we adjusted for age at diagnosis, current smoking, and surgery status. The analysis was carried out in STATA Version 15 (StataCorp).^43^

## Results

### Characteristics of the Study Sample

Our study population comprised 3695 white, non-Hispanic, adults: 2744 affected by IBD and 951 controls recruited by the IBDGC GRCs between 2003 and 2012. Out of the 2744 affected individuals, 55% carried the diagnosis of CD, 42.2% UC, and 2.8% IBD-U.

IBD patients were significantly younger (37.6 ± 15.8 vs 43.5 ± 14.5, *P* < .001), more often male (49.8% vs 43.4%, *P* < .001), and of Jewish ethnicity (13.9% vs 8.9%, *P* < .001) compared to controls. The median period from disease diagnosis to enrollment in the registry was 8 years (25th–75th percentile: 3–15 years). In our database, 28% of IBD patients were diagnosed before 1990, with 72% diagnosed between 1990 and 2011.

### Disease Expression and Determinant Factors in Affected Population

In our cohort, the average age at diagnosis significantly increased over time. Patients diagnosed post 1990 were significantly older at diagnosis than those diagnosed before 1990. The mean age for CD diagnosis was 22.7 vs 25.3 years in pre-1990 vs post-1990 era, 26.4 vs 31.9 for UC, and 24.0 vs 28.3 for IBD, respectively (*P* < .001 in all 3 groups) (Table 1). However, patients diagnosed before 1990 who were older at the time of diagnosis were less likely to be alive. They would have been older when the registry opened in 2003 and with each year of recruitment.

**Table 1:** Demographic, Clinical, and Genetic Characteristics of Affected Patients Pre-1990 and 1990+

Characteristics	Crohn’s disease		*P* value	Ulcerative colitis		*P* value	IBD		*P* value
	<1990	1990+		<1990	1990+		<1990	1990+	
	N = 372	N = 1136		N = 218	N = 940		N = 601	N = 2143	
Enrolment age, mean years ± SD	48.8 ± 11.4	31.3 ± 13.8	<.001	51.4 ± 12.4	37.5 ± 15.8	<.001	49.6 ± 11.9	34.2 ± 15.1	<.001
Diagnosis age, mean years ± SD	22.7 ± 8.5	25.3 ± 12.9	<.001	26.4 ± 10.9	31.9 ± 15.3	<.001	24 ± 9.6	28.3 ± 14.5	<.001
Male, n (%)	170 (45.7)	539 (47.4)	.56	112 (51.4)	505 (53.7)	.53	290 (48.3)	1077 (50.3)	.39
Jewish, n (%)	61 (16.4)	152 (13.4)	.015	40 (18.3)	114 (12.1)	<.001	103 (17.1)	278 (13)	<.001
Disease duration, mean years (range)	24 (20–31)	5 (2–10.5)	<.001	23 (20–28)	5 (2–9)	<.001	24 (20–30)	5 (2–10)	<.001
Family history, n (%)	150 (40.3)	362 (31.9)	.003	69 (31.7)	277 (29.5)	.53	223 (37.1)	668 (31.2)	.006
Smoking at diagnosis			<.001			.004			<.001
Nonsmoker	208 (55.9)	792 (69.7)		150 (68.8)	646 (68.7)		368 (61.2)	1484 (69.2)	
Current smoker	127 (34.1)	236 (20.8)		32 (14.7)	82 (8.7)		159 (26.5)	326 (15.2)	
Ex-smoker	32 (8.6)	96 (8.5)		32 (14.7)	207 (22)		65 (10.8)	315 (4.7)	
Missing	5 (1.3)	12 (1.1)		4 (1.8)	5 (.5)		9 (1.5)	18 (.8)	
Genetic risk score	4.8 (5.8)	4.2 (7.9)	.19	1.8 (1.3)	1.8 (1.5)	.39	2.7 (3.1)	2.4 (2.7)	.031
Surgery, n (%)			<.001			<.001			<.001
Yes	306 (82.3)	500 (44)		57 (26.1)	150 (16)		366 (60.9)	661 (30.8)	
Missing	3 (.8)	6 (.5)		1 (.5)	5 (.5)		4 (.7)	12 (.6)	
Location			<.001						
L1	97 (26.1)	314 (27.6)		-	-	-	-	-	
L2	29 (7.8)	171 (15.1)		-	-	-	-	-	
L3	180 (48.4)	512 (45.1)		-	-	-	-	-	
L4 only	.0 (0)	8 (.7)		-	-	-	-	-	
Perianal	63 (16.9)	123 (10.8)							
Missing	3 (.8)	8 (.7)		3 (1.4)	10 (1.1)	-	-	-	
Extent						.14			
E1	-	-		13 (2.2)	62 (6.6)	-	-	-	
E2	-	-		80 (36.7)	361 (31.2)	-	-	-	
E3	-	-		122 (56)	587 (62.4)	-	-	-	
Disease behavior			<.001						
B1	95 (25.5)	625 (55)		-	-	-	-	-	
B2	111(29.8)	231 (20.3)		-	-	-	-	-	
B3	154 (41.4)	265 (23.3)		-	-	-	-	-	
Missing	12 (3.3)	15 (1.32)		-	-	-	-	-	
Extraintestinal manifestations									
Arthritis			.04			.11			.004
Yes	78 (21)	185 (16.3)		30 (13.8)	96 (10.2)		110 (18.3)	292 (13.6)	
Missing	6 (1.6)	8 (.7)		7 (3.2)	10 (1.1)		13 (2.2)	19 (.9)	
Ankylosing spondylitis			.87			.20			.004
Yes	11 (3)	38 (3.3)		7 (3.2)	18 (1.9)		18 (3.0)	75 (2.7)	
Missing	8 (2.2)	12 (1.1)		7 (3.2)	12 (1.3)		15 (2.5)	25 (1.2)	
Uveitis/Episcleritis			1.00			.093			.23
Yes	19 (5.1)	58 (5.1)		9 (4.1)	20 (2.1)		28 (4.7)	78 (3.6)	
Missing	7 (1.9)	6 (.5)		4 (1.8)	10 (1.1)		11 (1.8)	16 (.7)	
Pyoderma gangrenosum			1.00			.71			.85
Yes	7 (1.9)	21 (1.8)		1 (.5)	11 (1.2)		8(1.3)	32 (1.6)	
Missing	10 (2.7)	6 (.5)		4 (1.8)	13 (1.4)		14 (2.3)	19 (.9)	
Primary sclerosing cholangitis			.56			.055			.17
Yes	5 (1.3)	11 (1.0)		14 (6.4)	33 (3.5)		19 (3.2)	46 (2.1)	
Missing	10 (2.7)	11 (1.0)		6 (2.8)	14 (1.5)		16 (2.7)	25 (1.2)	
Erythema nodosum			.16			1.00			.12
Yes	19 (5.1)	40 (3.5)		3 (1.4)	13(1.4)		22(3.7)	53 (2.5)	
Missing	8 (2.2)	8 (.7)		5 (2.3)	17 (1.8)		13 (2.2)	25 (1.2)	

Montreal classification for Crohn’s disease extent (L1: ileal, L2: colorectal, L3: ileocolonic, L4: upper gastrointestinal) and behavior (B1: inflammatory, B2: stricturing, B3: penetrating) and ulcerative colitis (E1: rectal, E2: left side colitis, E3: pancolitis).

SD, standard deviation.

CD patients compared to UC patients were younger at diagnosis (26.0 vs 30.9, *P* < .001), more frequently female (53.3% vs 47.0%, *P* = .005), and more frequently had a positive IBD family history (34.0% vs 29.9%, *P* = .014) (Table A1). CD patients were more likely to be active smokers than UC patients (*P* < .001). The most common CD site was ileocolonic (L3) (45.9%), and the most common disease behavior was inflammatory (B1) (47.7%). Twelve percent of patients had perianal disease.

CD patients had a significant change in disease site after 1990 (*P* < .001) primarily from an increase in colon-only disease site (15.1% vs 7.8%). They also were less likely to have penetrating disease behavior (B3) (Table 1). Median disease duration at the time of phenotyping was significantly shorter among patients diagnosed post 1990 than among those diagnosed before 1990 (5 years vs 24 years, *P* < .001). This factor may partly account for less-observed penetrating disease and less aggressive or severe disease post 1990. Among UC patients, 61.2% had pancolitis, with left colonic (31.2%) or proctitis-only disease (6.5%) being less frequent (Table A1). Disease site for UC did not change post 1990 with a similar proportion of patients with pancolitis present (Table 1).

Overall, 53.4% of CD patients had undergone surgery in comparison with only 17.9% of UC patients (*P* < .001) (Table A1). EIM of erythema nodosum and arthritis were significantly more frequent among CD than among UC patients while uveitis/episcleritis and primary sclerosing cholangitis were both more common in UC (*P* < .001).

#### Predictors for Age at Diagnosis

We assessed the predictors of age at diagnosis across all the 3 groups (CD, UC and IBD overall) using simple and multivariable linear regression models for “age at diagnosis” (Table 2). As far as innate risk factors, having a higher GRS was significantly associated with younger age at diagnosis for CD, UC, and IBD overall in univariate analyses but remained significant only for CD and IBD phenotypes in multivariate analyses. For each unit increase in GRS, the age at diagnosis decreased by a month (0.11 years). In addition, Jewish ancestry was associated with younger age at diagnosis for CD and IBD, but not UC, and only in multivariate analyses, after controlling for all other factors. Jewish ancestry patients were 1 year younger at diagnosis than non-Jewish ancestry patients (the estimate for age of diagnosis for CD using Jewish ancestry was 1.1 years, *P* = .012). Interestingly, family history of IBD was not associated with younger age at diagnosis in any analyses.

**Table 2:** Results of Univariable and Multivariable Linear Regression Models for Age at Diagnosis

Model	Crohn’s disease, N = 1508		Ulcerative colitis, N = 1158		Inflammatory bowel disease, N = 2744	
	Univariable	Multivariable	Univariable	Multivariable	Univariable	Multivariable
1990–1999 vs <1990	2.03^d^ [.67, 3.39]	12.97^d^ [12.12, 13.83]	4.740^d^ [2.75, 6.73]	13.80^d^ [12.81, 14.78]	3.564^d^ [2.383, 4.746]	13.52^d^ [12.88, 14.16]
>2000 vs <1990	2.948^d^ [1.73, 4.86]	19.94^d^ [19.09, 20.80]	5.970^d^ [3.95, 7.89]	20.84^d^ [19.89, 21.79]	4.825^d^ [3.69, 5.96]	20.62^d^ [20.01, 21.24]
Enrollment age	.57^d^ [.53, .60]	.83^d^ [.80, .86]	.75^d^ [.71, .78]	.92^d^ [.90, .94]	.67^d^ [.65, .70]	.882^d^ [.87, .90]
Jewish ethnicity	−.46 [−2.28, 1.37]	−1.1^b^ [−1.97, −.24]	−1.64 [−4.17, .89]	−1.01 [−1.97, .06]	−1.09 [−2.64, .46]	−1.06^c^ [−1.69, −.43]
IBD family history	−.4 [−1.71, .91]	−.15 [.67, .38]	−.53 [2.23, 1.18]	−.03 [−.61, .55]	−.67 [−1.76, .42]	- .131 [−.49, .23]
Current smoker^a^ vs nonsmoker	5.54^d^ [4.29, 6.78]	.5 [−.14, 1.13]	3.67^c^ [1.28, 6.07]	−.83 [−1.97, .31]	4.2^d^ [3.09, 5.31]	.21 [−.73, .32]
Ex-smoker^a^ vs nonsmoker	15.49^d^ [13.34, 17.65]	2.9^d^ [1.83, 3.98]	14.26^d^ [12.44, 16.08]	1.06^c^ [.46, 1.66]	16.03^d^ [14.71, 17.35]	1.95^d^ [1.41, 2.49]
Genetic risk score	−.13^d^ [−.21, .06]	−.11^b^ [−.20, .03]	−.61^b^ [−1.19, .04]	.09 [−.06, .24]	−.33^d^ [−.51, .15]	−.09^d^ [−.15, .02]

Data are presented as beta coefficient for the slope for each predictor and 95% confidence intervals.

a Referes to smoking status at diagnosis.

b *P* < .05.

c *P* < .01.

d *P* < .001.

CD and UC patients who “ever smoked” were older at diagnosis than in those who “never smoked” (data not shown). This same comparison was made by dividing smoking into either “current smoking” or “ex-smoking at diagnosis,” and each subcategory was significantly associated with age at diagnosis in univariate analyses. However, in multivariate analysis, only ex-smoking remained significantly associated with older age at diagnosis. The association of ex-smoking and older age at diagnosis (in multivariate analyses) was stronger for CD (β = 2.9, 95% CI 1.83–3.98) than for UC (β = 1.06, 95% CI 0.46–1.66, *P* < .001).

#### Smoking Status and IBD

We further explored the potential relationships of smoking, ex-smoking, and nonsmoking at diagnosis in the 2 different eras, noting that for the population of North America overall, smoking prevalence decreased after as compared to before 1990.^44^ For patients with either CD or UC, there was a statistically significant decline in the prevalence of *smoking* at diagnosis in both eras (Figure). The prevalence of *ex-smoking* at diagnosis increased amongst UC patients post 1990 and was greater in UC than in CD (Figure).

**Figure:**
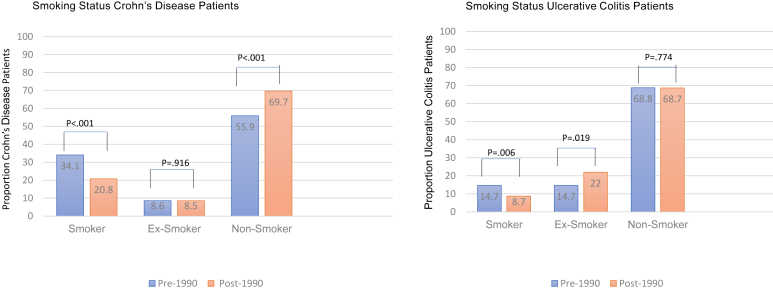
Smoking status in Crohn’s disease and ulcerative colitis before 1990 and after 1990.

While ex-smoking prevalence at diagnosis increased among UC patients post 1990, the frequency of *nonsmoking* at diagnosis remained identical between the 2 eras (Table 1) despite the overall trend for nonsmoking to increase in the North American population during the same periods. The opposite was true for CD: nonsmoking at diagnosis increased post 1990, whereas ex-smoking at diagnosis was essentially identical (Figure).

#### Genetic Risk Score

Univariate analysis for CD showed that a higher CD-GRS was significantly associated with diagnosis before 1990, younger age at diagnosis, Jewish ethnicity, IBD family history, and CD surgery. CD surgery demonstrated the strongest association with CD-GRS (Table 3). In multivariate analysis, all these factors remained significantly associated with CD-GRS except for diagnosis before 1990 vs post 1990.

**Table 3:** Genetic Risk Score: Simple and Multivariable Quantile Regression Models

Model	Crohn’s disease, N = 1508		Ulcerative colitis, N = 1158		Inflammatory bowel disease, N = 2744	
	Simple	Multivariable	Simple	Multivariable	Simple	Multivariable
Diagnosis y (1990+ vs <1990)	−.438^b^ [−.871, −.004]	−.097 [−.494, .301]	.068 [−.134, .270]	.03 [−.189, .25]	−.114 [−.292, .065]	.017 [−.174, .208]
Diagnosis age	−.016^c^ [−.027, −.004]	−.018^c^ [−.03, −.007]	−.0001 [−.003, .005]	−.003 [−.008, .003]	−.008^c^ [−.013, −.003]	−.007^c^ [−.120, −.002]
Jewish ethnicity	.923^b^ [.14, 1.70]	1.02^b^ [.42, 1.62]	.009 [−.122, .297]	.100 [−.129, .329]	.483^d^ [.31, .653]	.35^b^ [.082, .617]
Family history	.374^b^ [.016, .732]	.346^b^ [−.011, −.68]	−.0006 [−.161, .160]	.003 [−.163, .168]	.236^c^ [.081, .390]	.15 [−.021, .322]
Current smoker vs nonsmoking^a^	.049 [−.314, .413]	.113 [−.300, .527]	.095 [−.135, .325]	.159 [−.087, .404]	.089 [−.854, .264]	.145 [−.057, .347]
Ex-smoker vs nonsmoking^a^	−.053 [−.569, .464]	.145 [−.299, .589]	.056 [−.11, .22]	.115 [−.84, .315]	−.28^d^ [−.407, −.153]	.009 [−.207, .225]
Surgery vs no surgery	.673^d^ [.341, 1.006]	.681^d^ [.360, 1.002]	−.004 [−.191, .183]	.04 [−.156, .241]	.301^d^ [.135, .466]	.254^c^ [.089, .418]

Data presented as beta coefficient and 95% confidence interval in brackets. Beta coefficients can be interpreted as adjusted risk difference of GRS per unit increase in the independent variable.

a Refers to smoking status at diagnosis.

b *P* < .05.

c *P* < .01.

d *P* < .001.

For UC patients, neither simple linear nor multivariable analyses showed any of the evaluated factors significantly associated with UC-GRS.

For IBD overall, age at diagnosis, Jewish ethnicity, family history, ex-smoking, and surgery were all associated with IBD-GRS in univariate analyses. In multivariable analyses, age at diagnosis, Jewish ethnicity, and IBD surgery remained significantly associated with IBD-GRS, but IBD family history was no longer significant (Table 3).

As surgery is an outcome rather than an innate risk factor for IBD and considering that surgery frequency may be affected by greater follow-up in the pre-1990 than post-1990 subjects, we also performed multivariate analysis excluding surgery from the model (Table A2). For CD, we observed a borderline significant association between lower CD-GRS and diagnosis before 1990 (*P* = .58). For UC, results did not change. IBD-GRS showed associations with IBD family history and smoking (Table A2).

We also performed further multivariate analyses to clarify the complex effects of smoking status as it relates to the era of CD or UC onset and genetic risks (based on GRS), with age at diagnosis as a potential confounding variable (Table 4). For CD, being a smoker at diagnosis vs a nonsmoker at diagnosis was significantly more likely before 1990 and in patients with low GRS (*P* < .001). Being a smoker was also a significant determinant of an older age at diagnosis. For UC and IBD overall, being a smoker vs nonsmoker at diagnosis was more likely before 1990 and was associated with older age at diagnosis for IBD patients. No other variables showed significant independent associations for UC or IBD overall (CD, UC, and IBD-U combined).

**Table 4:** Results of Multivariable Generalized Linear Regression for Binomial Family for Smoking at Diagnosis, Adjusted for Era of Diagnosis, Diagnosis Age, and Genetic Risk Score

Variable	Crohn’s disease, N = 1491	Ulcerative colitis, N = 1149	Inflammatory bowel disease, N = 2717
1990+ vs <1990	−.153^b^ [−.204, −.102]	−.0648^a^ [−.116, −.0137]	−.123^b^ [−.162, −.0849]
Diagnosis age	.00732^b^ [−.00578, −.00886]	.000579 [−.00038, .00154]	.00232^b^ [.00145, .00318]
Genetic risk score	−.000759^b^ [−.0009, −.00062]	.0049 [−.0659, .0165]	.00251 [−.0042, .0047]

Data presented as beta coefficients and 95% confidence intervals in brackets. Beta coefficients can be interpreted as adjusted risk difference of smoking vs nonsmoking at diagnosis per unit increase in the independent variable.

a *P* < .05.

b *P* < .001.

## Discussion

Our study is a unique temporal overview of IBD disease expression and the contribution of risk factors for disease development in North America. It is perhaps the first study to examine differences in IBD features as well as known risk factors such as genetics, Jewish ethnicity, family history, and smoking in persons diagnosed before and during the contemporary era in North America before and after 1990, during which there has been greater suspicion of IBD and use of more-sensitive and routine procedures to make IBD diagnoses and when the incidence of IBD increased significantly. We observed that in our National Institutes of Diabetes, Digestion and Kidney Diseases IBDGC cohort (a cohort used in multiple studies to elucidate the genetics of IBD as well as other factors), features are similar to those of most cohorts^44^, ^45^, ^46^ with only a few exceptions.

### Genetic Risk

Our study elucidated the association of genetic risk with young onset and need for surgery among patients with CD and IBD. Compared with before 1990, patients diagnosed in the start of 1990 had a significantly lower CD-GRS (beta coefficient, −0.438, 95% CI −0.871 to −0.004) in univariate analysis but not in multivariable analysis (beta coefficient, −0.097, 95% CI −0.494 to 0.301) (Table 3). However, when we excluded surgery from multivariate analysis, the beta-coefficient decreased from −0.097 to −0.412 (95% CI −0.838 to 0.0137) with a trend toward significance (*P* = .058) suggesting that a lower genetic risk threshold may be permissible for disease to develop in more recent decades. This finding may be obscured by concurrent less-severe disease and decreased need for surgery. For UC, we observed none of the genetic risk-associated features. Our observation for UC is in contrast to the Cleynen et al^23^ report where younger onset was associated with a higher UC-GRS. Potential explanations are our smaller sample size and higher percentage of patients with more extensive and perhaps more severe disease than in the study by Cleynen et al. However, as suggested by prior studies on disease concordance for monozygotic twins, UC has less genetic influence than CD (15% vs 30% twin concordance).^47^

### Environment

Smoking at diagnosis was significantly more common among CD patients than among UC patients in our cohort. The opposite was true for ex-smoking. These differences in smoking and ex-smoking between the 2 IBD phenotypes remained significant and consistent, independent of the periods examined. However, mirroring the trend in the general population,^48^ smoking decreased significantly among both CD and UC patients post 1990 (Figure). This finding implies that for CD, there has been an increase in incidence, albeit with a concomitant decrease in exposure to this well-established environmental risk factor.^49^

Ex-smoking status was more prevalent in UC patients diagnosed post 1990. Therefore, the causal association of ex-smoking as a risk factor for this phenotype is concordant with the greater incidence of UC post 1990. This concordance may partly explain UC’s uptrending. Similar findings were reported in a systematic review of inception cohorts.^50^

Our most interesting finding may be that GRS-CD had a negative beta coefficient in multivariable analysis showing significant association with smoking at diagnosis in CD (Table 4), suggesting an important concept: When a person has the environmental risk factor for CD of smoking, one may only need a lower genetic risk (ie, GRS) to develop CD.

In the context of a decline in overall smoking among CD patients after 1990 and possibly a lower genetic risk contribution after than before 1990, but at a time when CD incidence increased, one might ask, “What are we missing in understanding the pathogenesis of CD?” Two possibilities are that CD incidence had not really changed but rather is being diagnosed much more frequently, and/or CD after 1990 may include more patients with less severe disease that may have gone undiagnosed in previous decades. Support for these hypotheses are that (1) nonsmoking is associated with less severe CD; (2) colon-only disease was significantly increased post 1990, concurrent with the routine utilization of colonoscopy and biopsy to evaluate chronic diarrhea and other symptoms of CD and noting that colon-only CD was shown by Cleynen et al to have lower genetic risk than either ileal-colonic or ileal-only disease^23^; and (3) there was significantly less CD surgery in our cohort post 1990 (44% vs 82%, *P* < .001), suggesting a more severe disease in CD patients diagnosed in the earlier decades. To normalize surgery for differences in follow-up between the cohorts, we examined surgery within 5 years of diagnosis. CD surgery remained significantly increased before 1990 despite this restriction (41.4 vs 32.4, *P* = .002). Although the degree of surgery may have been affected by the introduction of anti-tumor necrosis factor (TNF) therapy in 1998, this would have applied to only a part of the post-1990 cohort (ie, those recruited from 1999 to 2011), and its use would have been incremental during this period. Additionally, observational studies showed only a modest decrease, if any, in surgical rates during the early biologic era and primarily when aTNF biologics were used within the first years of diagnosis,^51^^,^^52^ a less common practice in the first decade of aTNF therapy when top-down therapy was still in the process of acceptance.^53^

A third and important consideration is that our study was limited in examination of environmental effects to that of smoking. However, over the decades leading up to the 1990s, there has been an increase in environmental factors or the “exposome” (eg, increased BMI, consumption of food detergents, nonsteroidal anti-inflammatory drug use and antibiotic use, and decreased infectious disease exposures including *Helicobacter pylori*) that may contribute to IBD onset and subsequent flares^54^^,^^55^ despite decreased smoking rates. Such exposures may modulate the composition or metabolic activity of the intestinal microbiota, thus indirectly modulating immune functions.^55^ Analysis of the exposome is complicated by the fact that individuals are always confronted with a mixture of exposures throughout their lifespan. Nonetheless, it will be important in future—perhaps prospective studies—to evaluate environmental IBD risk factors in addition to smoking in the context of genetic risks, ideally in prospective studies.

Our study has limitations. First, we studied subjects being cared for at tertiary referral centers, and therefore, our results may be subject to selection bias with patients tending to have more severe and refractory disease than population-based cohorts. However, in many instances, our population mirrored the characteristics of IBD population base cohorts.^52^, ^53^, ^54^ There will be a concern of volunteer bias in recruitment (and given the extent of genetic analysis, study subject volunteering for consensual genetic research will be a necessary limitation); however, each study center recruited all patients available, and although women tend to volunteer more than men, we only observed a slightly greater rate of CD in females (53.0%) and a greater rate of UC in males (53.3%), differences in sex mirroring population-based studies.^44^ We also had the expected percentage of Jewish population for the regions of recruitment (15%), and CD patients were younger at diagnosis than UC patients.^44^^,^^45^ Our UC patients, however, had a greater extensive disease site (61.2% of patients), which was the focus of recruitment in the first 2 years after the initiation of the registry, and this was true for both pre-1990 and post-1990 patients. Second, as noted, we did not have information on all suspected IBD risk factors and could not address the role of the microbiota in the epidemiology of the disease. Third, this is a cross-sectional study, not a longitudinal population-based study, and thus may not thoroughly answer the questions of trends in risk factors, and our study examining patients diagnosed prior to 1990 will necessarily be limited by survival bias. However, the large sample size reflecting different eras of diagnosis overcomes some of these limitations, and survival to at least the 1990s would be an inherent limitation in any sizeable study cohort that includes molecular genetic data.

## Conclusions

We still have much to discover regarding the driving factors and mechanisms of IBD. We expected lower genetic effects, as determined by GRS, to be observed for IBD, CD, and UC from the post-1990 era than the pre-1990 era. However, evidence for this was borderline and only when we excluded surgery in the model. However, we did observe that smoking was associated with a decreased GRS in patients that developed CD, validating a concept that when there is an increase in the presence of an environmental risk factor, a lower genetic risk may be required for disease to develop. This is parallel to that observed for reduced lung function and smoking: where being in the lowest decile of polygenic risk for reduced lung function but with 50+ pack-years of smoking exposure showed a similar effect as those in the highest decile of polygenic risk but having only 11–20 pack-years of smoking exposure.^56^ For UC, ex-smoking may account for some of the increase in UC incidence after 1990. Since the European ancestry population make-up is not known to have changed greatly (and we accounted for genetics of the high-risk Ashkenazi Jewish population), unaccounted environmental risk factors, in addition to increased diagnosis of mild and colon-only disease, likely underlie the increase in post-1990 IBD incidence, whether CD or UC. It will be important to uncover these risk factors and also determine the gene-environmental interactions that occur and explain the increase in IBD.

## Supplementary Material

Table A1

Table A2

## References

[1] Molodecky N.A., Soon I.S., Rabi D.M. (2012). Increasing incidence and prevalence of the inflammatory bowel diseases with time, based on systematic review. Gastroenterology.

[2] GBD 2017 Inflammatory Bowel Disease Collaborators (2020). The global, regional, and national burden of inflammatory bowel disease in 195 countries and territories, 1990-2017: a systematic analysis for the global burden of disease study 2017. Lancet Gastroenterol Hepatol.

[3] Siew N.C., Shi H.Y., Hamidi N. (2018). A worldwide incidence and prevalence of inflammatory bowel disease in the 21st century: a systematic review of population-based studies. Lancet.

[4] Dahlhamer J.M., Zammitti E.P., Ward B.W. (2016). Prevalence of inflammatory bowel disease among adults aged ≥18 years—United States, 2015. MMWR Morb Mortal Wkly Rep.

[5] Bernstein C.N., Shanahan F. (2008). Disorders of a modern lifestyle: reconciling the epidemiology of inflammatory bowel diseases. Gut.

[6] De Souza H.S., Fiocchi C. (2016). Immunopathogenesis of IBD: current state of the art. Nat Rev Gastroenterol Hepatol.

[7] Chang J.T. (2020). Pathophysiology of inflammatory bowel disease. N Engl J Med.

[8] Ramos G.P., Papadakis K.A. (2019). Mechanisms of disease: inflammatory bowel diseases. Mayo Clin Proc.

[9] Santos M.P.C., Gomes C., Torres J. (2018). Familial and ethnic risk in inflammatory bowel disease. Ann Gastroenterol.

[10] Halme L., Paavola-Sakki, Turunen U. (2006). Family and twin studies in inflammatory bowel disease. World J Gastroenterol.

[11] Moller F.T., Andersen V., Wohlfahrt J. (2015). Familial risk of inflammatory bowel disease: a population-based cohort study 1977-2011. Am J Gastroenterol.

[12] Ogura Y., Bonen D.K., Inohara N. (2001). A frameshift mutation in NOD2 associated with susceptibility to Crohn's disease. Nature.

[13] Hugot J.P., Chamaillard M., Zouali H. (2001). Association of NOD2 leucine-rich repeat variants with susceptibility to Crohn's disease. Nature.

[14] Jostins L., Ripke S., Weersma R.K. (2012). Host-microbe interactions have shaped the genetic architecture of inflammatory bowel disease. Nature.

[15] Liu J.Z., van Sommeren S., Huang H. (2015). Association analyses identify 38 susceptibility loci for inflammatory bowel disease and highlight shared genetic risk across populations. Nat Genet.

[16] Ellinghaus D., Jostins L., Spain S.L. (2016). Analysis of five chronic inflammatory diseases identifies 27 new associations and highlights disease-specific patterns at shared loci. Nat Genet.

[17] Parkes M., Barrett J.C., Prescott N.J. (2007). Sequence variants in the autophagy gene IRGM and multiple other replicating loci contribute to Crohn's disease susceptibility. Nat Genet.

[18] Yamazaki K., Umeno J., Takahashi A. (2013). A genome-wide association study identifies 2 susceptibility Loci for Crohn's disease in a Japanese population. Gastroenterology.

[19] Anderson C.A., Boucher G., Lees C.W. (2011). Meta-analysis identifies 29 additional ulcerative colitis risk loci, increasing the number of confirmed associations to 47. Nat Genet.

[20] Kenny E.E., Pe'er I., Karban A. (2012). A genome-wide scan of Ashkenazi Jewish Crohn's disease suggests novel susceptibility loci. PLoS Genet.

[21] Julià A., Domènech E., Chaparro M. (2014). A genome-wide association study identifies a novel locus at 6q22.1 associated with ulcerative colitis. Hum Mol Genet.

[22] Yang S.K., Hong M., Zhao W. (2014). Genome-wide association study of Crohn's disease in Koreans revealed three new susceptibility loci and common attributes of genetic susceptibility across ethnic populations. Gut.

[23] Cleynen I., Boucher G., Jostins L. (2016). Inherited determinants of Crohn's disease and ulcerative colitis phenotypes: a genetic association study. Lancet.

[24] Batura V., Muise A.M. (2018). Very early onset IBD: novel genetic aetiologies. Curr Opin Allergy Clin Immunol.

[25] Gordon H., Trier Moller F., Andersen V. (2015). Heritability in inflammatory bowel disease: from the first twin study to genome-wide association studies. Inflamm Bowel Dis.

[26] Ananthakkrishnan A.N., Berstein C.N., Iliopoulos D. (2018). Environmental triggers in IBD: a review of progress and evidence. Nat Rev Gastroenterol Hepatol.

[27] Herman S.M., Zaborniak K., Bernstein C.N. (2022). Insight into inflammatory bowel disease pathogenesis: is the answer blowing in the wind?. Inflamm Bowel Dis.

[28] Piovani D., Danese S., Peyrin-Biroulet L. (2019). Environmental risk factors for inflammatory bowel diseases: an umbrella review of meta-analyses. Gastroenterology.

[29] Mahid S.S., Minor K.S., Soto R.E. (2006). Smoking and inflammatory bowel disease: a meta-analysis [published correction appears in *Mayo Clin Proc*. 2007 82: 890]. Mayo Clin Proc.

[30] van der Sloot K.W.J., Weersma R.K., Alizadeh B.Z. (2020). Identification of environmental risk factors associated with the development of inflammatory bowel disease. J Crohns Colitis.

[31] M'Koma A.E. (2013). Inflammatory bowel disease: an expanding global health problem. Clin Med Insights Gastroenterol.

[32] Kaplan G.G. (2015). The global burden of IBD: from 2015 to 2025. Nat Rev Gastroenterol Hepatol.

[33] Coward S., Clement F., Benchimol E.I. (2019). Past and future burden of inflammatory bowel diseases based on modeling of population-based data. Gastroenterology.

[34] Keyashian K., Dehghan M., Sceats L. (2019). Comparative incidence of inflammatory bowel disease in different age groups in the United States. Inflamm Bowel Dis.

[35] Kaplan G.G., Ng S.C. (2017). Understanding and preventing the global increase of inflammatory bowel disease. Gastroenterology.

[36] Ananthakrishnan A.N., Kaplan G.G., Ng S.C. (2020). Changing global epidemiology of inflammatory bowel diseases: sustaining health care delivery into the 21st century. Clin Gastroenterol Hepatol.

[37] Rizello F., Spisni E., Giovanardi E. (2019). Implications of the westernized diet in the onset and progression of IBD. Nutrients.

[38] Ng S.C., Tang W., Ching J.Y. (2013). Incidence and phenotype of inflammatory bowel disease based on results from the Asia-Pacific Crohn's and colitis epidemiology study. Gastroenterology.

[39] Wei S.C., Lin M.H., Tung C.C. (2013). A nationwide population-based study of the inflammatory bowel diseases between 1998 and 2008 in Taiwan. BMC Gastroenterol.

[40] Shivashankar R., Tremaine W.J., Harmsen W.S. (2017). Incidence and prevalence of Crohn's disease and ulcerative colitis in Olmsted county, Minnesota from 1970 through 2010. Clin Gastroenterol Hepatol.

[41] Dassopoulos T., Nguyen G.C., Bitton A. (2007). Assessment of reliability and validity of IBD phenotyping within the National Institutes of Diabetes and Digestive and Kidney Diseases (NIDDK) IBD Genetics Consortium (IBDGC). Inflamm Bowel Dis.

[42] Brant S.R., Okou D.T., Simpson C.L. (2017). Genome-wide association study identifies African-specific susceptibility loci in African Americans with inflammatory bowel disease. Gastroenterology.

[43] Stata Corp (2017).

[44] Duricova D., Burisch J., Jess T. (2014). Age-related differences in presentation and course of inflammatory bowel disease: an update on the population-based literature. J Crohns Colitis.

[45] Nguyen G.C., Chong C.A., Chong R.Y. (2014). National estimates of the burden of inflammatory bowel disease among racial and ethnic groups in the United States. J Crohns Colitis.

[46] Vavricka S.R., Schoepfer A., Scharl M. (2015). Extraintestinal manifestations of inflammatory bowel disease. Inflamm Bowel Dis.

[47] Brant S.R. (2011). Update on the heritability of inflammatory bowel disease: the importance of twin studies. Inflamm Bowel Dis.

[48] (2014). The Health Consequences of Smoking—50 Years of Progress: a Report of the Surgeon General,” National Center for Chronic Disease Prevention and Health Promotion (US) Office on Smoking and Health.

[49] Papoutsopoulou S., Satsangi J., Campbell B.J. (2020). Review article: impact of cigarette smoking on intestinal inflammation-direct and indirect mechanisms. Aliment Pharmacol Ther.

[50] Thomas T., Chandan J.S., Li V.S.W. (2019). Global smoking trends in inflammatory bowel disease: a systematic review of inception cohorts. PLoS One.

[51] Peyrin-Biroulet L., Salleron J., Filippi J. (2016). Anti-TNF monotherapy for Crohn's disease: a 13-year multicentre experience. J Crohns Colitis.

[52] Ljung T., Karlén P., Schmidt D. (2004). Infliximab in inflammatory bowel disease: clinical outcome in a population-based cohort from Stockholm County. Gut.

[53] D'Haens G.R. (2010). Top-down therapy for IBD: rationale and requisite evidence. Nat Rev Gastroenterol Hepatol.

[54] Buck Louis G.M., Sundaram R. (2012). Exposome: time for transformative research. Stat Med.

[55] Rogler G., Vavricka S. (2015). Exposome in IBD: recent insights in environmental factors that influence the onset and course of IBD. Inflamm Bowel Dis.

[56] Kim W., Moll M., Qiao D. (2021). Interaction of cigarette smoking and polygenic risk score on reduced lung function. JAMA Netw Open.

