# Supplementary material for: Inflammatory Bowel Diseases Before and After 1990
Source: Gastro Hep Adv. 2022 Aug 10;2(1):22–32. doi: 10.1016/j.gastha.2022.08.001 (PMC9851382; doi:10.1016/j.gastha.2022.08.001)
Supplement: Table A1 [file mmc1.docx]

|  | | | | | |
| --- | --- | --- | --- | --- | --- |
| **Supplemental Table 1: Demographic, Clinical, and Genetic Characteristics of Affected Patients** | | | | |  |
|  | **Crohn’s Disease**  **N=1,508** | **Ulcerative Colitis**  **N=1,158** | **Inflammatory Bowel Disease**  **N=2,744** | **P-value** |  |
| **Enrollment age (mean± SD)** | 35.6 ±15.3 | 40.1 ± 16.1 | 37.6 ± 15.8 | <0.001 |  |
| **Diagnosis age (mean ± SD)** | 24.6 ± 12.0 | 30.9 ± 14.7 | 27.4 ± 13.7 | <0.001 |  |
| **Sex male, n (%)** | 709 (47.0) | 617 (53.3) | 1,367 (49.8) | 0.005 |  |
| **Jewish ethnicity, n (%)** | 213 (14.1) | 154 (13.3) | 13.9 (381) | 0.30 |  |
| **Family history IBD, n (%)^1^** | 512 (34.0) | 346 (29.9) | 32.5 (891) | 0.014 |  |
| **Smoking status, n (%)^2^** |  |  |  | <0.001^3^ |  |
| Non-smoker | 1,000 (66.3) | 796 (68.7) | 1,852 (67.5) |  |  |
| Smoker | 363 (24.1) | 114 (9.8) | 485 (17.7) |  |  |
| Ex-smoker | 128 (8.5) | 239 (20.6) | 380 (13.8) |  |  |
| Missing | 17 (1.1) | 9 (0.8) | 27 (1.0) |  |  |
| **GRS (mean ± SD)** | 4.3 (7.5) | 1.8 (1.5) | 2.5 (2.8) | n/a |  |
| **Surgery, n (%)** | 896 (53.4) | 207 (17.9) | 1,027 (37.4) | <0.001 |  |
| **Location, n (%)^4^** |  |  |  |  |  |
| L1 | 411 (27.3) | - | - |  |  |
| L2 | 200 (13.3) | - | - |  |  |
| L3 | 692 (45.9) | - | - |  |  |
| L4 | 8 (0.5) | - | - |  |  |
| Perianal | 186 (12.3) | - | - |  |  |
| Missing | 11 (0.7) | 13 (1.1) | - |  |  |
| E1 | - | 75 (6.5) | - |  |  |
| E2 | - | 362 (31.2) | - |  |  |
| E3 | - | 709 (61.2) | - |  |  |
| **Extra-gastrointestinal** | 383 (25.4) | 213 (18.4) | 613 (22.3) | <0.001 |  |
| PSC   Missing | 16 (1.1)  21 (1.4) | 47 (4.1)  20 (1.7) | 65 (2.4)  41 (1.5) | <0.001 |  |
| Pyoderma gangrenosum   Missing | 28 (1.9)  16 (1.1) | 12 (1)  17 (1.5) | 42 (1.5)  33 (1.2) | 0.11 |  |
| Erythema nodosum   Missing | 59 (3.9)  16 (1.1) | 16 (1.4)  22 (1.9) | 75 (2.7)  38 (1.4) | <0.001 |  |
| Uveitis/Episcleritis   Missing | 77 (5.1)  13 (0.9) | 29 (10.9)  17 (1.5) | 106 (3.9)  27 (1) | <0.001 |  |
| Arthritis   Missing | 263 (17.4)  14 (0.9) | 126 (10.9)  19 (19) | 402 (14.7)  32 (1.2) | <0.001 |  |
| Ankylosing spondylitis   Missing | 49 (3.2)  20 (1.3) | 25 (2.2)  19 (1.6) | 75 (2.7)  40 (1.5) | 0.19 |  |
| ^1^Any family history. ^2^Smoking status at enrollment. ^3^Standard analysis for a 3-category variable with mutually exclusive categories. ^4^Montreal classification for Crohn’s disease (L1: ileal, L2: colorectal, L3: ileocolonic, L4: upper gastrointestinal), behavior (B1: inflammatory, B2: structuring, B3: penetrating), and ulcerative colitis (E1: rectal, E2: left side colitis, E3: pancolitis). SD: standard deviation, IBD: inflammatory bowel disease, GRS: genetic risk score, PSC: primary sclerosing cholangitis. | | | | |  |
