# Supplementary material for: Inflammatory Bowel Diseases Before and After 1990
Source: Gastro Hep Adv. 2022 Aug 10;2(1):22–32. doi: 10.1016/j.gastha.2022.08.001 (PMC9851382; doi:10.1016/j.gastha.2022.08.001)
Supplement: Table A2 [file mmc2.docx]

| **Supplemental Table 2. Results of Simple and Multivariable Quantile Regression Models for Genetic Risk Score** | | | | | | |
| --- | --- | --- | --- | --- | --- | --- |
|  | **Crohn’s Disease** | | **Ulcerative Colitis** | | **Inflammatory Bowel Disease** | |
|  | Without Surgery  N=1470 | With Surgery  N=1478 | Without Surgery  N=1145 | With Surgery  N=1139 | Without Surgery  N=2700 | With Surgery  N=2685 |
| **Diagnosis year**  **(1990+ vs <1990)** | -0.412  [-0.838, -0.014] | -0.097  [-0.494, 0.301] | 0.01  [-0.208, 0.227 | 0.03  [-0.189, 0.25] | 0.003  [-0.210, 0.216] | 0.017  [-0.174, 0.208] |
| **Diagnosis age** | -0.019  [-0.032, -0.005] | -0.018**  [-0.03, -0.007] | -0.003  [-0.009, 0.003] | -0.003  [-0.008, 0.003] | -0.008  [-0.013, -0.003] | -0.007  [-0.120, -0.002] |
| **Jewish ethnicity** | 0.869**  [0.22, 1.518] | 1.02***  [0.42, 01.62] | 0.0995  [-0.108, 0.307] | 0.1  [-0.129, 0.329] | 0.336**  [0.88, 0.583] | 0.35*  [0.082, 0.617] |
| **Family history** | 0.4*  [0.05, 0.75] | 0.346*  [0.011, 0.68,] | 0.015  [-0.149, 0.179] | 0.003  [-0.163, 0.169] | 0.203*  [0.003, 0.46] | 0.15  [-0.021, 0.322] |
| **Current smoker vs. non-smoker** | 0.145  [-0.243, 0.532] | 0.113  [-0.300, 0.527] | 0.166  [-0.08, 0.412] | 0.159  [-0.087, 0.405] | 0.232*  [-0.854, 0.264] | 0.145  [-0.057, 0.347] |
| **Ex-smoker vs non-smoker** | -0.118  [-0.656, 0.421] | 0.145  [-0.299, 0.589] | 0.104  [-0.09, 0.299] | 0.115  [-0.84, 0.315] | 0.00  [-0.245, 0.140] | 0.009  [-.207, 0.225] |
| **Surgery vs**  **no-surgery** | - | 0.681***  [0.360, 1.002] | - | 0.043  [-0.156, 0.241] | - | 0.254**  [0.089, 0.419] |
| **Intercept** | 2.939***  [2.430, 3.448] | 2.939***  [2.430, 3.448] | 1.436***  [1.178, 1.694] | 1,403***  [1.137, 1.669] | 1.739***  [1.503, 1.974] | 1.634***  [1.404, 1.865] |
| Presented are beta coefficient for the slope for each predictor, and 95% confidence intervals 95% in brackets, ^*^ *p* < 0.05, ^**^ *p* < 0.01, ^***^ *p* < 0.001 | | | | | | |
